# Chromosome genome assembly and annotation of the yellowbelly pufferfish with PacBio and Hi-C sequencing data

**DOI:** 10.1038/s41597-019-0279-z

**Published:** 2019-11-08

**Authors:** Yitao Zhou, Shijun Xiao, Gang Lin, Duo Chen, Wan Cen, Ting Xue, Zhiyu Liu, Jianxing Zhong, Yanting Chen, Yijun Xiao, Jianhua Chen, Yunhai Guo, Youqiang Chen, Yanding Zhang, Xuefeng Hu, Zhen Huang

**Affiliations:** 1 The Public Service Platform for Industrialization Development Technology of Marine Biological Medicine and Product of State Oceanic Administration, Fujian Key Laboratory of Developmental and Neural Biology, College of Life Sciences, Fujian Normal University, Fuzhou, Fujian China; 2 School of Computer Science and Technology, Wuhan University of Technology, Wuhan, Hubei China; 3 Fujian Key Laboratory of Special Marine Bio-resources Sustainable Utilization, Fujian Normal University, Fuzhou, Fujian China; 4 Fisheries Research Institute of Fujian, Xiamen, Fujian China; 5 Fujian Fishery Technical Extension Center, Fuzhou, Fujian China; 6 National Institute of Parasitic Diseases, Chinese Center for Disease Control and Prevention; Key Laboratory of Parasite and Vector Biology, Ministry of Health, Shanghai, 200025 China

**Keywords:** Conservation genomics, Sequencing, Ichthyology, Genome

## Abstract

Pufferfish are ideal models for vertebrate chromosome evolution studies. The yellowbelly pufferfish, *Takifugu flavidus*, is an important marine fish species in the aquaculture industry and ecology of East Asia. The chromosome assembly of the species could facilitate the study of chromosome evolution and functional gene mapping. To this end, 44, 27 and 50 Gb reads were generated for genome assembly using Illumina, PacBio and Hi-C sequencing technologies, respectively. More than 13 Gb full-length transcripts were sequenced on the PacBio platform. A 366 Mb genome was obtained with the contig of 4.4 Mb and scaffold N50 length of 15.7 Mb. 266 contigs were reliably assembled into 22 chromosomes, representing 95.9% of the total genome. A total of 29,416 protein-coding genes were predicted and 28,071 genes were functionally annotated. More than 97.7% of the BUSCO genes were successfully detected in the genome. The genome resource in this work will be used for the conservation and population genetics of the yellowbelly pufferfish, as well as in vertebrate chromosome evolution studies.

## Abstract (summary)

**Table:** 

Measurement(s)	whole genome sequencing assay • transcription profiling assay • sequence_assembly • sequence annotation
Technology Type(s)	DNA sequencing • RNA sequencing assay • genome assembly • bioinformatics analysis
Sample Characteristic - Organism	Takifugu flavidus
Sample Characteristic - Environment	aquatic environment

Machine-accessible metadata file describing the reported data: 10.6084/m9.figshare.10008740

## Background & Summary

The yellowbelly pufferfish (FishBase ID: 24266), *Takifugu flavidus*, is an economically and ecologically important fish species in coastal regions of East Asia, including the East China Sea, Yellow Sea and Bohai Bay^1,2^. The yellowbelly pufferfish is also a temperate bottom fish that exhibits only short-distance seasonal migration^3^. The yellowbelly pufferfish is caught and cultivated as a delicious fish species with high market value^2,4,5^. However, due to environmental deterioration and overfishing, the wild populations of the species have declined in the last decade^6,7^. Additionally, a low survival rate in artificial breeding has greatly limited the development of the marine aquaculture of the yellowbelly pufferfish^8,9^. Previous studies of the yellowbelly pufferfish have mainly focused on behavioural^1^, morphological and growth characteristics, temperature and salinity effects on embryos and larval development^9^, and molecular marker development^10^. A genome of *Takifugu flavidus* was published in 2014^11^; however, this genome was a fragmented draft with contig and scaffold N50 lengths of 2.7 kb and 305.7 kb, respectively. A high-quality reference genome of the yellowbelly pufferfish could facilitate and prompt conservation genetics research and investigation of the molecular mechanisms of important economic traits of the species.

The genomes of pufferfish have also played an important role in studies of vertebrate genome evolution due to the compactness of genus *Takifugu* genomes^12–15^. Previous studies have shown that the number of repetitive elements in pufferfish is significantly reduced^14,15^. The genome of *Takifugu rubripes*, the other pufferfish in genus *Takifugu*, exhibits conserved linkages with humans, implying the preservation of chromosomal segments from the common vertebrate ancestor^13–15^. Another pufferfish genome, that of *Tetraodon nigroviridis*, was the second pufferfish genome reported^14^. Although the yellowbelly pufferfish genome has been published, the genome was constructed using short reads from SOLiD next-generation sequencing, and the sequences have not been assembled at the chromosomal level^11^. The elucidation of genomic evolution among pufferfish species, and comparison with other vertebrates such as humans, will require further chromosomal genome assemblies for pufferfish species.

**Fig. 1:**
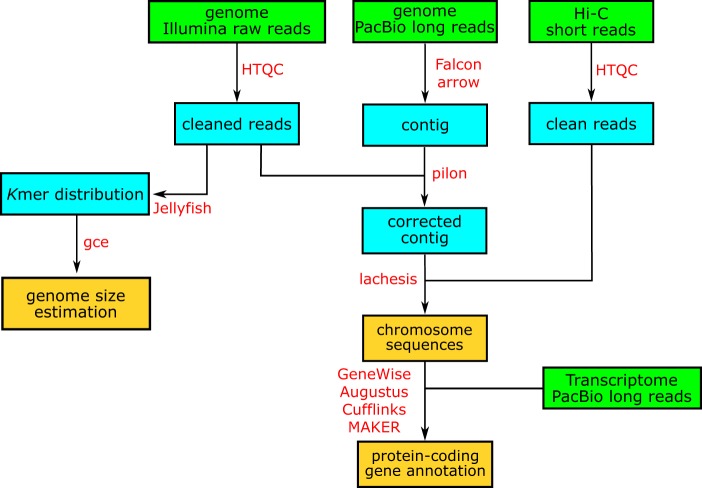
The work flow used for the yellowbelly pufferfish genome assembly and annotation in this work. The panes with green, cyan and yellow represent the input sequencing data, intermediate files and final outputs, respectively. Bioinformatics software is highlighted in red along the work flow.

In this work, we applied a combined strategy involving Illumina, PacBio and Hi-C technologies to generate sequencing data for chromosomal genome construction for the yellowbelly pufferfish (Fig. 1). More than 98% of BUSCO genes were detected, and the contig and scaffold N50 lengths reached 4.4 and 15.7 Mb, respectively, demonstrating the outstanding completeness and sequence continuity of the genome. A total of 29,416 protein-coding genes were predicted in the assembled genome, and more than 95% of those genes were successfully functionally annotated. We believe that the chromosomal genome assembly constructed in this work will not only be valuable for ecology, conservation and aquaculture studies of the yellowbelly pufferfish but will also be of general interest in the evolutionary investigation of teleosts and vertebrates.

## Methods

### Sample collection

**Fig. 2:**
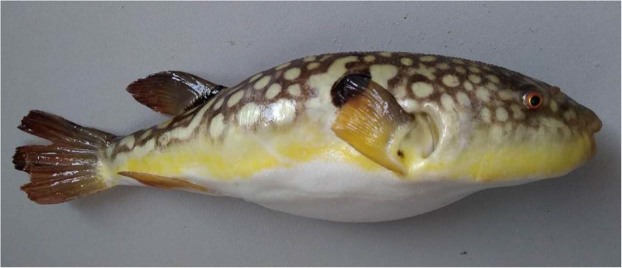
A picture of the yellowbelly pufferfish used in the genome sequencing and assembly.

A female yellowbelly pufferfish (Fig. 2), reared in the fish breeding centre of Fujian Normal University in Fuzhou City of Fujian Province was used for genome sequencing and assembly. Fresh white muscle, eye, skin, gonad, gut, liver, kidney, blood, gall bladder and air bladder tissues were collected and quickly frozen in liquid nitrogen for one hour. White muscle tissues were used for DNA sequencing for genome assembly, while all tissues were used for transcriptome sequencing.

### DNA and RNA sequencing

Genomic DNA from white muscle tissue was extracted using the standard phenol/chloroform extraction method for DNA sequencing library construction. The integrity of the genomic DNA molecules was checked using agarose gel electrophoresis. Both the Illumina HiSeq X Ten platform and the PacBio SEQUEL platform were applied for genomic sequencing to generate short and long genomic reads, respectively. For the Illumina X Ten platform (San Diego, CA, USA), a paired-end library was constructed with an insert size of 250 base pairs (bp) according to the protocol provided by the manufacturer. As a result, 44 Gb (~120X coverage of the estimated genome size, Table 1) of accurate short reads were generated, which were further cleaned using the HTQC utility^16^. Adapter sequences and reads with more than 10% N bases or low-quality bases (≤5) were filtered from the sequencing data. After filtering, 41.8 Gb (~110X, Table 1) of cleaned data were retained for the following analysis. To obtain sufficient sequencing data for genome assembly, we constructed two 20 kb DNA libraries using the extracted DNA and the standard Pacific Biosciences (PacBio, Menlo Park, CA) protocol, and fragments were selected using the Blue Pippin Size-Selection System (Sage Science, MA, USA). The library was sequenced using the PacBio SEQUEL platform. After removing adaptors, we obtained 27.2 Gb subreads (~73X, Table 1) for genome assembly. The genomic sequencing data used for subsequent genome assembly are summarized in Table 1.

**Table 1:** Sequencing data used for the yellowbelly pufferfish genome assembly.

Library resource	Sequencing platform	Insert size	Raw data (Gb)	Sequence coverage (X)
genome	Illumina HiSeq X Ten	250 bp	41.8	110
genome	PacBio SEQUEL	20 kb	27.2	73
Hi-C	Illumina HiSeq X Ten	250 bp	50.7	132
transcriptome	PacBio SEQUEL	0.6–3 kb	13.1	—

Note that the sequence coverage values were calculated based on the genome size estimated by the *K*mer-based method.

We also performed RNA sequencing to generate transcriptome data for gene model prediction. To include as many tissue-specific transcripts as possible, multiple tissues were collected, as indicated in the Sample Collection section. TRIzol reagent (Invitrogen, USA) was used to separately extract RNA from all of the collected tissues, including white muscle, ocular, skin, gonad, intestine, liver, kidney, blood, gall bladder and air bladder tissues. RNA quality was checked with a NanoDrop ND-1000 spectrophotometer (Labtech, Ringmer, UK) and a 2100 Bioanalyzer (Agilent Technologies, CA, USA). Then, RNA molecules were equally mixed for transcriptome sequencing on the PacBio SEQUEL platform. First, cDNA was prepared using the SMARTer PCR cDNA Synthesis Kit (Clontech) from 1 μg of purified RNA. The Iso-Seq libraries were constructed from the BluePippin (Sage Science, MA, USA) size-selected cDNA with a size range of 0.6–3 kb according to the PacBio SEQUEL library construction protocol. Two SMRT flow cells were used for long-read transcriptome sequencing, and the resulting data used for gene prediction are summarized in Table 1.

### *De novo* assembly of the yellowbelly pufferfish genome

**Fig. 3:**
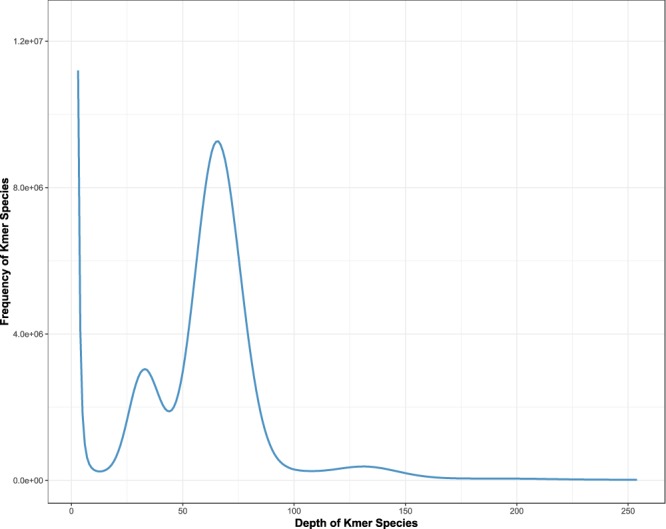
The 17-mer count distribution for the genome size estimation. Note that the peaks around the depths of 33, 66 and 132 represent the heterozygous, homozygous and repeated *K*mers, respectively.

For the Next Generation Sequencing (NGS) short reads, the *K*mer-based method^17^ was used to perform genome survey analysis to estimate the genome size, heterozygosity and repeat content of the yellowbelly pufferfish genome. We counted the number of each 17-mer with Jellyfish^18^, and the frequency distribution is plotted in Fig. 3. The yellowbelly pufferfish genome size was then estimated from the frequency distribution to be 377 Mb.

The Falcon package^19^ was used to assemble the yellowbelly pufferfish genome with PacBio long reads, using the parameters length_cutoff = 10 kb and pr_length_cutoff = 8 kb. The procedures of long-read self-correction, corrected read alignment, sequence graph construction, and contig assembly were performed in Falcon. To correct random sequencing errors in the Falcon output, two steps of genome sequence polishing were applied: we first used arrow^20^ to polish the genome using long sequencing data, and two rounds of polishing using NGS short reads were then applied with Pilon^21^. Finally, we obtained a final contig assembly of 366 Mb with a contig N50 length of 4.4 Mb (Table 2).

**Table 2:** Assembly statistics for the yellowbelly pufferfish.

Content	Length				Number			
	Contig (Mb)		Scaffold (Mb)		Contig		Scaffold	
	new	old	new	old	new	old	new	old
Total	366.26	278.46	366.28	366.28	1,117	376,565	867	3,226
Max	12.82	0.046	28.84	2.8	—	—	—	—
Number > =2 kb	—	—	—	—	1,115	23,662	867	3,146
N50	4.4	0.0011	15.7	0.37	28	64,775	10	251
N90	0.4	0.0003	11.7	0.055	127	241,187	21	1,198

Note that the term contig here refers to the continuous sequences obtained after the Hi-C-data-based chromosome construction. Note that “new” represents the genome assembled in the present work and that “old” refers to the genome published in 2014.

To evaluate the quality of the assembled genome, the completeness and accuracy were assessed via BUSCO analysis and short-read mapping. The completeness of the assembled yellowbelly pufferfish genome was assessed by using BUSCO v3.0^22^ with the vertebrata_odb9 database. We found that 95.7% and 2.7% of 2,586 BUSCO genes were completely and partially BUSCO genes were detected in the genome. We also aligned NGS short reads to the genome and found that more than 98.5% of the reads were reliably aligned, showing a high mapping ratio for the short-read sequencing data.

### Chromosome construction using interaction information from Hi-C data

In this work, we applied the Hi-C technique for chromosome construction for the yellowbelly pufferfish. Although the Hi-C technique was first introduced to quantify genome-wide interactions^23^, the method exhibits suitability for chromosome assembly and has been successfully applied in many genomic projects^24^. In our study, we used 0.2 ml of blood from the same individual used for genome sequencing for Hi-C library construction and sequencing using the same method as in a previous study^25,26^. From the Hi-C library sequencing, approximately 50.7 Gb of data were generated (Table 1). The sequencing reads were mapped to the polished yellowbelly pufferfish genome with Bowtie 1.2.2^27^. We independently aligned the two read ends to the genome and only selected the read pairs for which both ends were uniquely aligned to the genome. The hiclib Python library^28^ and a previously reported method^24^ were applied to filter the Hi-C reads, and the interaction frequency was quantified and normalized among contigs. Lachesis^29^ with default parameters was then applied to cluster contigs with the agglomerative hierarchical clustering method using the interaction matrix between sequences. Among the 169 million read pairs generated from Hi-C sequencing, 59 million read pairs (34.9%) provided valid interaction information for chromosome assembly. As a result, the contigs from the yellowbelly pufferfish were successfully clustered into 22 groups, which were further ordered and oriented into chromosomes. Finally, 271 contigs were reliably anchored on chromosomes, accounting for 95.9% of the total genome. The contig and scaffold N50 values reached 4.4 and 15.7 Mb (Tables 2 and 3), respectively, providing the first chromosomal genome assembly for the yellowbelly pufferfish.

**Table 3:** Summary of the assembled chromosomes of the yellowbelly pufferfish.

Chr	Chr length (bp)	Contig number	Gene number
Chr1	28,838,366	15	2,171
Chr2	19,632,357	11	1,243
Chr3	19,136,632	13	1,361
Chr4	18,781,179	28	1,639
Chr5	18,395,123	16	1,444
Chr6	16,875,900	15	1,440
Chr7	16,703,359	13	1,189
Chr8	16,202,710	8	1,268
Chr9	15,776,270	8	1,063
Chr10	15,676,631	7	1,215
Chr11	15,654,207	13	1,091
Chr12	15,631,021	10	1,269
Chr13	15,542,920	11	1,272
Chr14	15,503,328	17	1,341
Chr15	15,463,098	11	1,395
Chr16	14,247,604	12	1,103
Chr17	13,381,174	14	986
Chr18	13,174,367	19	1,324
Chr19	12,605,058	6	993
Chr20	12,303,402	10	991
Chr21	11,708,235	5	868
Chr22	9,947,389	9	747

### Gene model prediction and functional annotations

Repeat elements were annotated in the yellowbelly pufferfish before gene model annotation. We applied Tandem Repeat Finder (TRF)^30^, LTR_FINDER^31^, PILER^32^ and RepeatScout^33^ for the *ab initio* prediction of repeat elements in the genome. Thereafter, RepeatMasker and RepeatProteinMask (http://www.repeatmasker.org) were used to search the genome sequences for known repeat elements, with the genome sequences used as queries against the Repbase database^34^. The repetitive element annotations are listed in Table 4.

**Table 4:** Repetitive element annotations in the yellowbelly pufferfish.

	No. of TEs	Length (bp)	% of total TEs	% of genome
**Total repeat fraction**	300,773	60,927,544	100	16.63
**Class I: Retroelement**	77,720	29,919,159	49.11	8.17
**LTR retrotransposon**	20,782	10,394,098	17.06	2.84
Ty1/Copia	865	227,841	0.37	0.06
Ty3/Gypsy	7,440	3,670,541	6.02	1.00
Other	12,477	6,495,716	10.66	1.77
**Non-LTR retrotransposon**	51,417	18,060,948	29.64	4.93
LINE	37,274	16,042,878	26.33	4.38
SINE	14,143	2,018,070	3.31	0.55
**Unclassified retroelement**	5,521	1,464,113	2.40	0.40
**Class II: DNA transposon**	39,742	11,514,017	18.90	3.14
**TIR**				
CMC[DTC]	3,477	365,955	0.60	0.10
hAT	8,606	3,318,856	5.45	0.91
Mutator	406	110,998	0.18	0.03
Tc1/Mariner	9,293	3,015,854	4.95	0.82
PIF/Harbinger	2,035	672,257	1.10	0.18
Other	6,632	1,014,243	1.66	0.28
**Helitron**	74	12,208	0.02	0.00
**Tandem repeats**	194,185	19,801,588	32.50	5.41
**Unknown**	1,412	870,602	1.43	0.24

Gene annotation was performed on the repetitive-element-masked genome. A combined strategy of homology-based, *ab initio* and transcriptome-based gene prediction methods was used. Protein sequences of *Astyanax mexicanus*, *Danio rerio*, *Gadus morhua*, *Ictalurus punctatus*, *Oryzias latipes*, *Takifugu rubripes*, *Tetraodon nigroviridis* and *Oreochromis niloticus* were downloaded from Ensembl^35^. Proteins from the closely related fish species were mapped to the yellowbelly pufferfish genome using TBLASTN^36^. The alignments were joined with Solar, and GeneWise^37^ was used to predict the exact gene structure of the corresponding genomic regions. Augustus^38^ was also used for the *ab initio* prediction of genes in the repeat-masked genome. Finally, the full-length transcriptome sequences generated from PacBio sequencing were aligned to the genome using the TopHat package^39^, and gene structure was predicted using Cufflinks^40^. All gene models were merged, and redundancy was removed by MAKER^41^, leading to a total of 29,416 protein-coding genes.

The NCBI non-redundant protein (nr) database and the SwissProt database with an E-value threshold of 1e-5 were used for the functional annotation of the protein-coding genes using BLASTX and the BLASTN utility^42^. Functional ontology and pathway information from the Gene Ontology (GO) and the Kyoto Encyclopedia of Genes and Genomes (KEGG) databases was assigned to the genes using Blast2GO^43^. Ultimately, 28,017 genes (95.24% of the total) of the yellowbelly pufferfish were functionally annotated (Table 5).

**Table 5:** The statistics of functional annotation of protein-coding genes.

Database	Number	Percent (%)
Nr	27,859	94.7
GO	16,533	56.2
KEGG	27,700	94.2
SwissProt	23,881	81.2
At least one database	28,017	95.2
Total	29,416	

Note that “at least one database” here refers to genes with at least one hit in multiple databases.

## Data Records

The sequencing dataset and genome assembly were deposited in public repositories. The previous genome assembly can be accessed under accession number AOOT01000000 in NCBI^44^. The genomic Illumina sequencing data, genomic PacBio sequencing data, transcriptomic PacBio sequencing data, and the genomic Hi-C sequencing data were deposited as SRP162225 in NCBI^45^. The final chromosome assembly and genome annotation were submitted to NCBI Assembly with accession number RHFK00000000^46^. The functional annotation files are also available at figshare^47^.

## Technical Validation

The quality of the DNA and RNA molecules and libraries used for genomic sequencing and transcriptome sequencing was validated before sequencing. The extracted DNA spectrophotometer ratios (SP) were 260/280 ≥1.6 for both Illumina and PacBio sequencing. DNA samples >2 μg and 20 μg were used for Illumina and PacBio sequencing, respectively. The concentration and quality of the total RNA were evaluated using a NanoVue Plus spectrophotometer (GE Healthcare, NJ, USA). RNA samples with a total RNA amount ≥10 μg, RNA integrity number ≥8, and rRNA ratio ≥1.5 were used to construct the sequencing library.

**Fig. 4:**
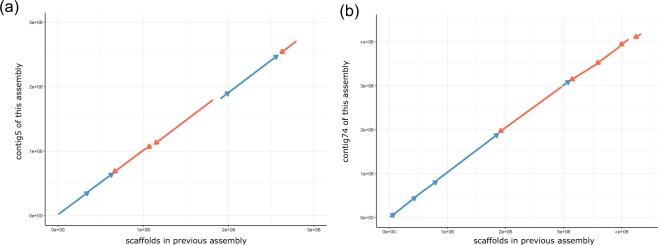
Two examples of the alignment of scaffolds from the previous genome assembly to our new yellowbelly pufferfish genome assembly. (**a**) Alignments on contig5 in the new genome. (**b**) Alignment on contig74 in the new genome. The X axis represents the scaffolds from the previous genome, and the Y axis represents the contig sequences assembled in this work. The straight and reverse alignments of the scaffold sequences are shown in blue and red, respectively.

To validate our genome assembly, we compared the new genome to the previous genome. The new genome contained significantly fewer ambiguous bases (0.02 Mb) than the previous genome (67.9 Mb), but the size (366 Mb) of the new genome was approximately 20 Mb larger than that of the previous genome (346 Mb). Considering the estimated genome size of 377 Mb determined from the *K*mer-based method, our new genome exhibited high completeness compared to the previous genome. The contig and scaffold N50 values of the newly assembled genome were almost 4,000 and 50 times higher than those of the previous genome, indicating a remarkable improvement in the sequence continuity of our assembly. We attributed the completeness and the continuity of the new genome to the application of PacBio long reads in the genome assembly. To further validate the improved continuity, we aligned genome fragments to our new genome with the NUCmer utility and found that more than 76% of the contigs were reliably mapped to the new genome with alignment ratios greater than 95%. Figure 4 provides two examples of alignments of genome sequences from the previous genome to our new assembly, showing that our new genome has significantly improved sequence continuity compared to the previous version.

## Usage Notes

The contig sequences were assembled into chromosomes using interaction information from Hi-C sequencing data; therefore we used 100 bp to represent the unknown gap sizes among contigs in the chromosome sequences.

## Data Availability

No specific code or script was used in this work. All commands used in the processing were executed according to the manual and protocols of the corresponding bioinformatics software.
